# Editorial Notes

**Published:** 1910-08

**Authors:** 


### EDITORIAL DEPARTMENT.

### EDITORIAL NOTES.

Some Remarkable and Rare New Books.—Funk & Wagnalls Company, the great publishers of New York, have sent us five new books—thus early in the season, an earnest of what is to follow later, and have requested us to give them an early review. The weather is so dreadfully hot that I am afraid a review will not keep, unless I put it in cold storage (and there isn't room there, as I have to occupy it myself), so I publish elsewhere notices sent me by the publishers. They are better and more effective than any I could write—even if I had time or inclination to read the books, with the thermometer at 104° in the shade. They are timely, however, for the doctors who would keep up with all that is new and valuable in the practice (I do not have to practice), and will make excellent summer reading while one is in camp or on vacation.

Funk & Wagnalls Company write us as follows:

We are sending you the latest and most up-to-date editions of these important works, and we hope that you will give them special attention.

Of "Syphilis" we are sending you the latest edition, just published embodying many alterations and amplifications not appearing in the earlier editions.

"Radiumtherapy" is an entirely new book and is the latest and most authentic on a subject that is now receiving much attention in the medical profession.

"The Diagnosis of Smallpox" has just been published by us and covers its subject most thoroughly and admirably.

As we have not published large editions of these most costly specialized works, only a very limited number can be sent out for review; and these are going to those few medical journals which, in our opinion, are the most important in their field.

See notices under "Books."

---

The Importance of Pediatrics and the Position of Surgicai Pediatrics to the College Curriculum.—The importance of pediatrics as a study in our medical schools and recognition of the surgical diseases of children as a department of study was among the admirable features of the report on curriculum presented at the meeting of the Association of American Medical Colleges held in Baltimore in March. The report placed the minimum number of hours to be devoted to pediatrics at 150, which is an increase of 50 per cent over the present requirement of the Association, and yet is a very modest share of the 4000 hours of the clinical years (the third and fourth years) of the college course. Dr. H. D. Arnold, of Boston, was chairman of the subcommittee on curriculum for the clinical years, and Dr. S. W. Kelley, of Cleveland, member representing pediatrics. The report goes on to say: "The allowance for pediatrics is intended to include instruction in exanthemata. In many other ways medicine and pediatrics overlap. Useless repetition can only be avoided by a proper understanding between the teachers of these two subjects, and a certain elasticity should be allowed a school for the purpose of assigning time to one subject or the other according to where the borderland subjects can be best taught. In the same way pediatrics and surgery touch and overlap. In one subject or the other surgery peculiar to children should receive attention. Valuable suggestions in relation to the teaching of pediatrics will be given in an appendix to this report.

---

IT MAKES US FEEL GOOD to get such evidences of appreciation as this. Sharp & Dohme, in remitting, write:

"We take this opportunity to express the hope that you will enjoy your summer outing and to assure you that S. & D. thoroughly appreciate your invariable courtesy and most excellent advertising service."

---

DEATH OF MRS. DEERING J. ROBERTS.—The JOURNAL is pained to note the death of this excellent woman—the life-mate of our old and greatly esteemed friend, Dr. Deering J. Roberts, of Nashville, Dean of the *Medical Press*. We beg to extend to him our sympathy. Mrs. Roberts was born in Adair county, Ky., January 10, 1844, and was, therefore, 66 years of age. Before her marriage she was Miss Rachael Lavina Breeding, daughter of Robert M. and Rachael Breeding. She was married to Dr. Deering J. Roberts January 27, 1867. During practically all of the forty-three years of her married life she lived in Nashville, where she was held in the highest regard by all who knew her.

Mrs. Roberts is survived by her husband and six children, John,

Harry, Albert, Robert and Deering J. Roberts, Jr., and Mrs. R. E. Turner. She is survived also by four brothers, G. B., W. M., and R. G. Breeding, all of Kentucky, James Breeding, of Iowa, and one sister, Mrs. John Royce.

---

Another Martyr to Medical Science.—Dr. M. K. Kassabian, of Philadelphia, died in that city July 12th ult., from the effects of the X-Rays, which he constantly used. He was a zealous searcher for effective methods of combating disease, and had an unbounded faith in the curative properties of the X-Rays. He had suffered years from the mysterious effects of this agency—was badly burned on hands and face, but he persisted and finally fell a victim to the enthusiasm. The announcement of his early death, following immediately on the publication by the Lippincott Company of a new revised edition of his great work on Electro-Therapeutics and the Roentgen Rays, comes as a great shock to the medical world.

---

Dr. J. J. Dial, of Sulphur Springs, Texas, one of the Old Guard ("the Old Guard never surrenders")—one who, like yours truly, stands for the old-time aristocracy of medicine—how be it, he is a member of the Mixed Board of Examiners, favored us with a call last week, looking as hale and handsome as ever. He has just finished a long course of post-graduate instruction under Murphy, Ochsner et al., and is ready now to tackle anything that comes along—medical or surgical.

